# Health workers’ disrespectful and abusive behaviour towards women during labour and delivery: A qualitative study in Durban, South Africa

**DOI:** 10.1371/journal.pone.0261204

**Published:** 2021-12-14

**Authors:** Sphindile Mapumulo, Lyn Haskins, Silondile Luthuli, Christiane Horwood

**Affiliations:** Centre for Rural Health, Howard College Campus, University of KwaZulu-Natal, Durban, South Africa; University of Cape Town, SOUTH AFRICA

## Abstract

**Background:**

A high prevalence of disrespectful and abusive behaviour by health workers towards women during labour and delivery has been widely described in health facilities, particularly in Africa, and is a worldwide public health concern. Such behaviours are barriers to care-seeking, and are associated with adverse outcomes for mothers and newborns. This paper reports experiences of disrespectful care among informal working women in three public health facilities in Durban, South Africa.

**Methods:**

A qualitative longitudinal study was conducted among a cohort of informal working women recruited during pregnancy in two clinics in Durban. The study comprised a series of in-depth interviews conducted at different time points from pregnancy until mothers had returned to work, followed by focus group discussions (FGDs) with cohort participants. We present data from participatory FGDs, known as ‘*Journey with my Baby’*, conducted at the end of the study, during which women’s experiences from pregnancy until returning to work were reviewed and explored. Thematic analysis was used with NVIVO v12.4.

**Results:**

Three *‘Journey with my Baby’* FGDs were conducted with a total of 15 participants between March and October 2019. Many participants narrated experiences of disrespectful behavior from nurses during labour and childbirth, with several women becoming very distressed as a result. Women described experiencing rudeness and verbal abuse from nurses, lack of privacy and confidentiality, nurses refusing to provide care, being denied companionship and being left unattended for long periods during labour. Women described feeling anxious and unsafe while in the labour ward because of the behaviour they experienced directly and observed other patients experiencing. Such experiences created bad reputations for health facilities, so that women in the local community were reluctant to attend some facilities.

**Conclusion:**

Disrespect and abuse continues to be a serious concern in public health facilities in South Africa. We challenge the health system to effectively address the underlying causes of disrespectful behavior among health workers, initiate robust monitoring to identify abusive behavior when it occurs, and take appropriate actions to ensure accountability so that women receive the high-quality maternity care they deserve.

## Introduction

High rates of maternal and neonatal mortality, particularly in low income settings, remain a global public health concern as reflected in the United Nations Sustainable Development Goals for health and wellbeing [1]. Interventions to improve the quality of care during labour and delivery are important to improve outcomes for women and babies [2]. Good quality of care may often be narrowly constructed as the provision of correct clinical treatment, but quality of care is a complex concept that comprises more than just adherence to treatment guidelines. Other important constructs of quality include interpersonal aspects of care, such as communication, joint decision-making and patient satisfaction, and these aspects of quality also influence outcomes [3–5].

Disrespectful and abusive behavior by health workers (HWs) towards women in labour and during delivery has been highlighted as a major public health concern in recent years [6, 7], and has been shown to be a common practice within healthcare facilities in many settings globally [8–10]. Disrespectful and abusive behavior has most often been described in low- and middle-income countries, but there are also reports of overmedicalization, unnecessary procedures including episiotomy and cesarean sections in many high income countries [11]. Aspects of disrespectful care that are commonly reported in low income countries include unfriendly provider attitudes [12], poor communication with providers [3], women not being well informed about their care or receiving care without their consent [3, 13], abandonment of care [13], lack of privacy [7] and in some cases physical, psychological and verbal abuse of women in labour and during delivery [3, 13, 14]. Such practices are a major barrier to uptake of maternal health services [15], are an important driver of poor quality of care, and are associated with increased maternal and neonatal mortality [16].

Bowser and Hill [6] identified seven categories of disrespect and abusive behavior that women reported experiencing during childbirth, comprising physical abuse, non-consented care, non-confidential care, non-dignified care, discrimination based on specific patient attributes, abandonment of care, and detention in facilities. A number of factors can perpetuate disrespect and abuse, including individuals and communities who normalize these behaviours during childbirth, lack of enforcement of national laws and policies that address disrespectful care, lack of governance and leadership, lack of supervision and accountability mechanisms in health care facilities, shortage of human resources and lack of professional development opportunities for HWs [6].

In response to concerns about disrespect and abuse, the White Ribbon Alliance led a global initiative to promote respectful maternal care and issued seven universal rights of childbearing women [17], based on international human rights instruments such as Universal Declaration of Human Rights [17]. In addition, the World Health Organization (WHO) issued a statement that “Every woman has the right to the highest attainable standard of health, which includes the right to dignified, respectful health care”, calling for health systems to be organised and managed to ensure that women’s sexual and reproductive health and human rights are respected [18].

Disrespectful and abusive behaviors in maternity care are particularly widespread in many African settings [3], including in Kenya where the prevalence ranges from 20% to 78% [19], and in Nigeria where this was found to be 98% [8]. Concerns about disrespect and abuse have been reported in labour wards in South Africa, particularly in public health facilities, with the first published reports dating back to 1998 [20]. The SA government has addressed this at national level by ratifying a number of international human rights instruments that guarantee women’s rights, including the Convention on the Elimination of All forms of Discrimination against Women (CEDAW) [21]. In addition, the right of access to quality reproductive and maternal health care is enshrined in the South African constitution [22], and the latest SA Guidelines for maternity care emphasise the requirement to treat women with respect at all times, as well as women’s rights to privacy and companionship during labour [23]. In this paper we describe the current situation with regards to labour ward care in three facilities in South Africa, based on FGDs with groups of informal workers.

## Methods

The data presented is part of a larger prospective longitudinal mixed methods study conducted among women informal workers, to explore the relationship between child care and informal work. Participants were followed up from pregnancy until the mother returned to work or when the child reached one year of age. The methodology of the main study is described elsewhere [24]. Data were collected through a series of interviews undertaken at different time points over the follow up period, followed by a series of participatory focus group discussions (FGDs) conducted with groups of cohort participants. In this paper we present qualitative data from three participatory FGDs exploring women’s experiences of pregnancy, childbirth and caring for a young child over the entire follow-up period, known as *Journey with My Baby* (S1 File).

### Study setting

The study was conducted in two municipal wards on the outskirts of Durban, a large metropolitan city in KwaZulu-Natal, South Africa. Both wards are densely populated with 37 000 and 35 000 inhabitants respectively. While these wards have recently experienced an increase of informal economic activities resulting from economic development [25], average annual household income is low in both wards (R29 400; USD1800), unemployment is high at 41.5% and 32.2% and there are a high number of female-headed households: 40.2% and 48.7% [26].

Maternal care is provided through a district health system and women attend antenatal care at their nearest primary health care clinic. Antenatal, labour, delivery and postnatal care is provided free of charge in all public health facilities. In the Durban area maternal mortality ratio (MMR) was 102.4 per 100 000, and neonatal death in facility was 11.5 per 1000 live births in year 2018/19 [27]. The MMR in South Africa (119/100 000) is lower than many African countries including Zimbabwe (458/100 000), Tanzania (529/100 000) and Nigeria (917/100 000), reflecting South Africa’s status as a high middle-income country. However, several African countries have successfully reduced their MMR to lower levels, including Cape Verde (58/100 000), and Mauritius (61/100 000), and South Africa has higher MMR than other upper middle-income countries like Brazil (59/10000) [28].

### Recruitment and sampling

Participants were recruited in two large primary health care clinics, one in each of the two urban wards during the last trimester of pregnancy. Eligibility criteria included being an informal worker for more than 6 months, being 18 years or older and 32–38 weeks pregnant. The study defined informal workers as either an employer whose business was not tax registered, an employee without a contract or unemployment insurance fund (UIF), or an own account worker. Domestic workers who carried out domestic or childcare work in private households, were considered informal workers in this study regardless of whether they had contracts or contributed towards UIF. Women were not eligible to participate if they were working less than three days a week or planning to change the location after delivery.

A structured screening tool was used by two trained field workers to determine eligibility, and all eligible women were asked to participate. All women went through a three-phase recruitment process prior to enrolling to the cohort. Twenty-four women were enrolled in this study.

### Data collection

The *Journey with My Baby* FGDs were the final data collection activity for cohort participants. During the FGD women were asked to highlight significant moments they experienced from the time they found out about the pregnancy until the present time. Participants were given charts, marking pens and sticky notes and were instructed to draw a line across the middle of the chart and divide the line into months to tell the story of what happened at each time point. All women were asked to present their charts to the whole group. After each presentation, follow up questions were asked by facilitators and participants were given the opportunity to ask each other questions.

FGDs were conducted in the local language (IsiZulu) by two experienced qualitative researchers, trained to masters level (SL) and to honours level (SM). Researchers were both female and were fluent isiZulu speakers. The FGDs were conducted in offices at the University of KwaZulu-Natal.

### Data analysis

FGDs discussions were transcribed verbatim, translated into English, and quality controlled by researchers before the start of data analysis. Using an inductive analytic approach, transcripts were read by three coding researchers, major themes were identified and a coding framework was developed. Thematic analysis was then undertaken using NVIVO v12.3. Meetings were held frequently between the coding researchers to discuss the progress of the analyses and to identify any further emerging themes to be added to the coding framework.

### Ethical consideration

Ethics approval was obtained from the University of KwaZulu-Natal Humanities and Social Sciences Research Ethics Committee (HSSREC) (HSS/0319/018), the KZN Department of Health (HRKM235/18) and the World Health Organisation Ethics Committee (ERC 0003101). Written inform consent were provided by all participants who participated in the FGD. An amount of R150 (US$ 10,54) was given to participants to compensate them for their time away from work and R50 (US$ 3,51) for transport fee. Participants were given pseudonym to maintain anonymity and confidentiality.

## Results

Three FDGs were conducted on the topic of ‘*Journey with my Baby*’ between March and October 2019. In total, fifteen women (six, five and four per group) participated in the FGDs. One of the major themes that emerged from the data was women’s experiences during labour and childbirth. Among the 15 FGD participants, eight women described aspects of abusive or disrespectful care during childbirth, two women described positive child birth experiences, and five participants mentioned the childbirth experience in neutral terms.

We limit the results reported in this paper to the stories from eight women who experienced disrespectful or abusive care, both directed at themselves personally or directed at other patients in the ward. Demographic characteristics of participants are shown in Table 1. A few of the women who reported poor care became very distressed and emotional when relating their experiences of childbirth. Some participants wept openly and in one FGD the discussion was suspended for a short period to allow participants time to compose themselves. Women’s stories are summarised in Table 2.

**Table 1:** Demographic characteristics of participants.

Women’s age	n = 8
20–29	5
30 and above	3
Relationship status	
In a relationship and living with the partner	3
In a relationship and not living with the partner	4
Married	1
Highest grade passed	
Secondary school: grade 8 to grade 11	4
Completed schooling: grade 12	4
First time pregnancy	
Yes	1
No	7
Income per month	
Less than R1000	3
R1000-R3000	4
More than R3000	1

**Table 2:** Summary of stories of disrespect and abusive care experienced by participants.

Mother number	Place of birth (two hospitals & one clinic)	Lack of privacy and confidentiality	Rudeness and verbal abuse	Neglectful care: refusal to provide care	Denied birth companionship	Left unattended
M01	Hospital	Overheard nurses informing another woman that her baby had just died during birth.	Observed nurses shouting and scolding other women admitted in the labour ward		Participant did not bring companion	Participant reported that nurses were talking among themselves and did not pay attention to patients
		Became very anxious when she saw the dead baby wrapped up while she was still in labour.				
M02	Hospital	Overheard everything that was happening to other women in the labour ward	Observed other women being shouted at by a nurse	A nurse refused to assist the participant in fixing her bed which was broken and unstable	Participant did not bring companion	Participant heard the nurse leaving a woman in labour unattended because she was crying
M03	Hospital	While waiting in labour ward to be admitted, participant saw another woman giving birth alone without the nurses being present.		Participant was admitted in labour and put into a dirty bed (blood stained). When she complained the nurse told her to clean the bed herself.	Participant did not bring companion	Participant was left unattended, nurse asked the participant to call her when she feels that the baby is coming.
M04	Clinic			After the participant told the nurse that she feels like pushing, the nurse told the participant to get off bed and unpack baby’s clothes. Baby was delivered while she was still unpacking clothes.	Participant did not bring companion	
M05	Clinic	Observed both a mother and baby who died while she was on the labour and postnatal wards.	Needed assistance but when she asked for it, the nurse shouted at her telling her to stop calling them because they had just started their shift.	Participant asked the nurse for water to drink and was told to get water herself.	Participant brought her own mother to support her during labour but was told by the nurses that the mother cannot go into the labour ward.	Participant stated that the nurses were not attentive to herself and other women during labour. They spent most of their time talking among themselves
M06	Hospital				Participant wanted to be with her husband during birth but nurses stated that men are not allowed in the labour ward due to lack of privacy	
M07	Hospital		Participant complained that the nurses were shouting at her.		Participant wanted to be with the father of the child but he was denied access to labour ward.	Participant reported that when women in labour cry nurses do not attend them.

The behaviors that women particularly highlighted were rudeness from nurses, lack of privacy, ill-treatment, being denied a birth companion, and being neglected or abandoned during childbirth. We present these experiences based on the framework for PCMC which comprises three domains: dignity and respect; communication and autonomy; supportive care [16].

### Dignity and respect

HWs should treat women with dignity and respect, by treating them in a polite and friendly manner, and respecting their privacy and confidentiality. Women reported that it was the nurses who failed to treat them with dignity and respect during their time on the labour ward.

*It is the nurses that treat you badly*. *The doctors treat the patients well. I do not understand why that is the case (M07)*.

### Rudeness and verbal abuse

Verbal abuse of women by nurses during labour was commonly reported by participants who experienced it either directly towards themselves or indirectly by observing other women being abused during labour. The women who did not experience such treatment themselves, nevertheless described how being in an unsupportive environment, where they were fearful of being treated badly, had an effect on their labour.

*I was emotional because of hearing things that were happening on other beds*. *I do not want to lie (M02)*.

Women reported that nurses were shouting or spoke harshly to them during labour and delivery. When women were crying because of labour pains, nurses would yell at them telling them to stop crying. In addition, women who were not giving birth for the first time reported more verbal abuse from the nurses, because they were expected to be able to cope better with the labour, having experienced it before.

*If you are not giving birth for the first time they ask you why you are crying as if you have never given birth before (M07)*

### Lack of empathy

Nurses also showed lack of respect and empathy in how they spoke to women during labour. Participants reported that nurses were heard scolding women who were crying or distressed during labour or delivery. Nurses did not acknowledge the women’s distress but rather treated them with contempt. None of the women mentioned receiving any pain relief during labour.

*I heard others crying and screaming and I heard the responses that the nurses were giving to them*…. *Women get abused there*. *There was a lady that was crying and you could hear that she was running out of breath*. *The nurse said “hey you must tell me when the head has come out*. *Leave me alone”*. *(M02)*

Nurses also showed lack of empathy when there were complications during the delivery. In one case a woman described overhearing nurses speaking to the mother of a baby who had recently died and who was refusing further treatment.

*They were shouting to one another “the baby passed on (died) a long time ago*; *come and sign here to state that we will not be able to clean you”. (M01)*

### Lack of privacy

Lack of privacy within the healthcare facility was a reoccurring theme among participants. Nurses often breached the patients’ right to private care. Some women reported that while admitted to the labour ward, they could see and hear other women giving birth. This contributed to the stress and anxiety of women during labour. In this case a participant describes seeing a woman give birth without assistance while she herself was being admitted in labour.

*So, what I observed at [Hospital name] is that I saw a mother delivering a child on her own. We were still waiting (to be admitted) … I saw a woman give birth on her own*. *I saw the child coming out of her (M03)*

Many participants reported that they themselves did not receive care and were not able to give birth in a private space, out of sight and hearing of other patients. Lack of privacy contributed to stress and anxiety among women who were still in labour waiting to give birth.

*Her [dead] baby was wrapped there; she was sitting there. That was very hurtful because I thought maybe my baby was no more (had died) because I had never experienced such pain in my life*. *Maybe the reason why it is so painful is because my baby was dead, but it was just normal pain that every person experiences. (M01)*

### Confidentiality

It was common for nurses to talk loudly about women’s health status in front of other patients without respect for confidentiality. While in the labour ward, women would hear disturbing news such as a stillbirth or the death of a baby during delivery.

*I did not like the way they broke the news that the baby was no more (had died); the way the nurse spoke, I mean I even heard it*. *It would have been better if she took the lady aside and told her that this is what has happened; I would not have heard when I was so far away. (M01)*

### Communication and autonomy

Provisions in the domain of communication relate to HWs introducing themselves to women, addressing the mother by name and involving her in the decisions about her care. In terms of autonomy HWs should explain any procedures, obtain consent for procedures and answer the mother’s questions. Women in our study did not mention any of these aspects and it appeared they did not expect to be treated in this way. However, from the stories the women told, it was clear that communication did not reach the requirements for good communication and respecting autonomy. The woman below describes how nurses withheld care from those women who were distressed or complaining in order to punish them and make them keep quiet. Some of the nurses refused to attend women unless they stopped crying.

*When I went to give birth, the nurse was shouting and I could not understand why she was shouting at me because I was in a place where I could cry and I was there to seek help but she was just shouting at me….When they see that you are crying a lot they do not attend to you*. *They attend to you when you are calm. They come to ask if you are alright but if you are crying they do not attend to you at all. (M07)*

Women reported that communication was poor, when nurses spoke harshly women felt frightened of them, and as a result, women complied by being quiet and they avoided asking questions because they feared getting a harsh response.

*There is a lot of abuse that happens there. Sometimes you end up taking wrong decisions because of the way that you have been treated at the clinic…*. *sometimes you [are] even afraid to ask [for] a small thing because you are afraid of the answer that you will get there. You will regret why you even asked. (M02)*

### Supportive care

#### Women were denied birth companionship

Some women reported that they wished to have a birth companion to support them during labour and delivery, but this was refused despite, in some cases, there being signs in the ward informing them that birth companions were allowed.

*I was with my mother*. *I left [home] in the morning with my mother because she came when she realised that I was about to deliver because I live alone*. *She came to support me at that time. I went with her to [clinic name]. She was told that she cannot go in. I told her to go back home. (M05)*

This was stressful for women and their family members, including one example where the father of the child planned and wanted to be with the woman when giving birth, but he was refused entry. Women felt particularly alone and unsupported because nurses did not give them the support they needed. One woman suggested that the nurses did not allow birth companions because of their own unacceptable behaviour.

*I think they [nurses] want to avoid videos being taken and that the father of the child will talk about what he had seen [in the labour ward]*. *We are usually in pain in the labour ward so you will not pay much attention to most things there. (M03)*

Another woman noted that fathers may have been excluded from the labour ward to protect other women’s privacy, but still felt that if fathers were not allowed, women should be allowed to have an alternative female person to support them through labour while they are in pain.

*My first opinion is that hospitals that refuse access to fathers in the labour ward must allow at least one person to be there by your side while you are giving birth*…*You might expect that there should be someone close to you, maybe a nurse that will be able to check on you because you are in pain and you are crying. If they allow people to come into the labour ward at least you will have someone next to you comforting you no matter the amount of pain that you will be in. (SM07)*

#### Women were refused supportive care

Neglectful care included examples of behaviour where nurses refused to help or support women during labour. One participant who was booked to deliver at the district hospital because of a previous premature delivery and neonatal death, stated that when she started experiencing pain she went to the clinic and was sent home by nurses who claimed that she was not yet due for delivery, but later at night she was admitted to hospital and delivered her baby.

*I went there [clinic] and told them [nurses] that I have menstrual pains that I do not understand. They [nurses] told me that my baby is still far; at 21:00 I felt my back burning up and had the urge to go to the toilet*. *My mother told me I could not go to the toilet; she said I must go to hospital because the baby was coming*. *On the [same] day I had gone to the clinic I went to [hospital name]. When I got to [hospital name] I found other doctors who were not there during the day. They asked why they [clinic nurses] sent me away because I should have been admitted earlier and I said I did not know. (M08)*

During labour, women reported asking for assistance from nurses and there were several examples where participants reported that nurses refused to help them. One participant asked a nurse to help her fix the bed she was lying in which seemed to be unstable but the nurse refused and told the participant to fix the bed herself.

*One (part of the bed) goes this direction and the other goes that direction. When you ask for help and ask her (the nurse) to put it back together she will tell you to do it yourself*. *She does not care that you are in pain. You will do it yourself. She says that if you leave that thing, the child will fall (off the bed) and you will say it is their fault*. *She then leaves. (M02)*

Another participant stated that the nurses put her in a dirty bed, with bloodstains on the sheets. When she complained that the bed was messy, nurses told the participant to clean the bed herself.

*They gave me a bed that had blood in it*. *The sheets had blood*. *I asked why the bed had blood and they said I must clean it myself (M03)*

#### Women were left unattended

Several women stated that they were left unattended for long periods during labour. Although the nurses came regularly to check women’s progress in labour, they were otherwise left alone with no nurses in sight for long periods. Nurses asked the women to inform them when the child wants to come out. One participant described seeing a woman giving birth alone without assistance, and later she herself gave birth while the nurse was still preparing herself for the delivery.

*I could feel that the child was no longer in my belly but was near my bladder. The nurse said “please call me when you feel the head” because I tried to do this [gesturing] and then I felt that I was no longer going to touch my vagina but something else, the child’s hair*. *While she was putting on her gloves and doing everything I pushed the child out. When the child came out I was no longer feeling any pain. (M03)*

#### Poor care led to a lack of trust in the health facility

Two women described observing situations where a baby died, including in one case where both the mother and the baby died. These deaths occurred in the same health facility but on different occasions. Such stories created a bad reputation for health facilities causing women to avoid using them. One woman reported that in future she will not seek health care from this particular facility.

*Going to the clinic bothers me. As I have alluded, I am going to stop going there and go elsewhere. Maybe it will be better than [clinic name]*. *I am fed up with it. (M05)*

One woman reported that before labour she was scared of a particular hospital because of its bad reputation and opted to travel and go to another facility for her delivery. However, when she saw a woman losing her baby in her preferred facility, she wanted to be transferred to the hospital she had previously avoided.

*I have never had a bad experience at [hospital]*; *I was just scared to go there but that day when I went into labour I wanted to go to [hospital] because of what was happening at [clinic]. (M01)*

#### Poor infrastructure

Labour wards had poor infrastructure, which contributed to a lack of privacy. In particular, beds were close to each other. Nurses used curtains to create privacy, but women could hear and sometimes see what was happening behind the curtains. Poor infrastructure was also blamed for women being not allowed to bring their birth companion because there was a lack of privacy for other patients.

*His [child’s father] aim was to be present when I deliver because there are signs at the hospital that people can go in, but when he wanted to go in they say men are not allowed inside. It is only women that are allowed*. *They say it is because the beds are like this [gesturing close together] and when you give birth he will see everything that is happening. They said men are not allowed to go inside. (M06)*

**Fig 1:**
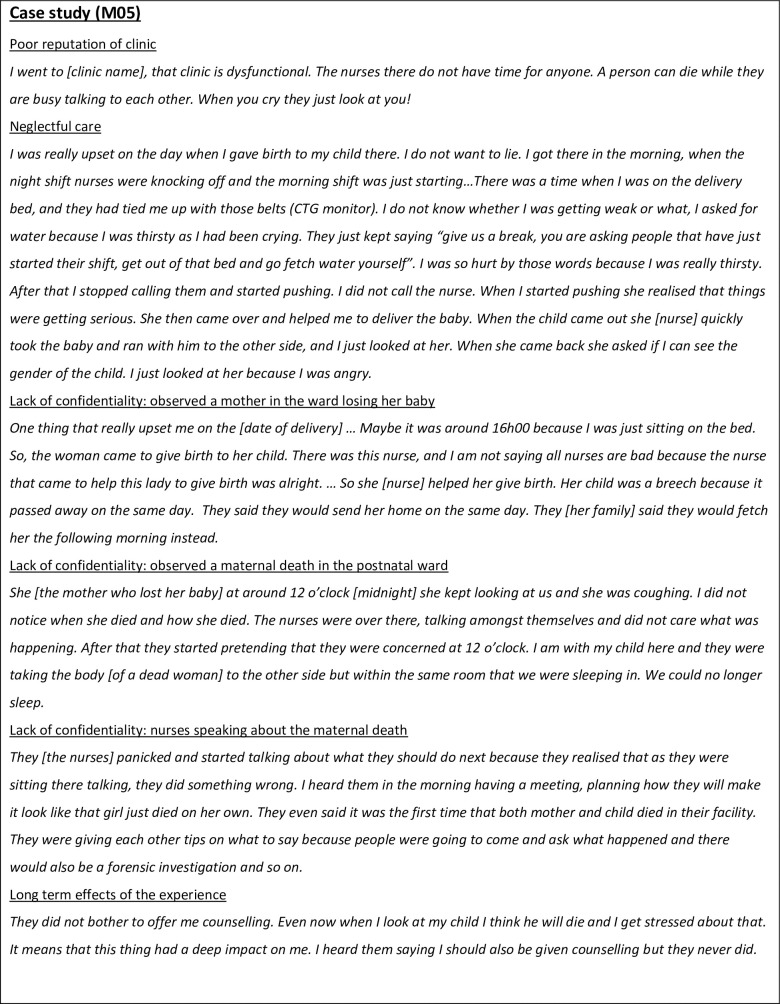
One woman’s story.

There was one woman who experienced a large variety of different types of disrespectful care, as told in her story of delivering in her local public health clinic (Fig 1).

## Discussion

This study reports women’s stories about their birth experiences in three public health facilities in South Africa. Women described stories of distress caused by rudeness, verbal abuse and lack of empathy from nurses, lack of privacy and confidentiality, abandonment of care, and absence of birth companions. Women in pain during labour were yelled at so they became frightened to ask questions, were refused assistance with basic supportive care, and were left unattended for long periods. In addition, some women described the trauma of watching other women giving birth without assistance, and even witnessing maternal and newborn deaths. Nurses on the labour ward used the power and status of their positions to belittle and dehumanise the women in their care. As a result, women felt degraded and were anxious and fearful in an environment that was supposed to make them feel comfortable and safe. Although disrespect and abuse has been reported in South Africa a number of times over the past two decades [3, 20, 29, 30], our study shows that little or nothing has changed in public health facilities.

Our findings echo findings from other African studies that report verbal abuse, including being scolded, mocked or threatened, as a common form of disrespect [14, 29] together with poor communication, lack of privacy and neglectful care [3, 31, 32]. However, we did not document incidences of physical abuse including slapping, pinching or restraining women, that have been described in other settings [13, 33]. Verbal abuse and neglect appeared to be normalized and institutionalized in our setting, with nurses openly and loudly voicing their abuse, similar to findings in other settings [34]. Our findings contrast to high income countries where the abuse profile is different, frequently involving unnecessary medical procedures, which can have long term complications and affect womens’ future childbearing decisions [11, 35]. It has been suggested that abuse of women in labour reflects a societal gender imbalance and could be considered an extension of gender-based violence in broader society [34]. However, in our study perpetrators were nurses and were almost always women themselves, so this behavior may reflect abuse they themselves experience at home, and that the abuse may be a compensation for lack of power in other aspects of their lives [20]. Gender inequalities are endemic in South Africa [36, 37].

Disrespectful and abusive practices in labour wards in the South African context are deep-rooted and complex given the history of gender and racial inequality [38]. These practices are a mix of individual abuse, structural configurations that promote degrading practices, and HW’s demeaning attitudes towards women. In addition, abusive maternity care should be seen as a component of societal devaluation of women and girls, as well as a normalization of violence against women in South Africa [38]. The SA constitution strongly supports women’s rights [22], and the SA government has ratified several international and local human rights tools requiring government structures to respect, protect and fulfil the rights of women and girls to health, equality and non-discrimination, including their sexual and reproductive rights [39]. This is reflected in the current SA guidelines for maternity care which state ‘*Treat all women in labour with respect and courtesy*. *Address the woman by her name*. *Ensure privacy and always perform intimate examinations behind screens*, *with a chaperone if needed*. *Allow family and friends to provide companionship during labour’* [23]. Our findings support other reports in demonstrating that these guidelines are poorly implemented [29, 30, 40]. The health system in South Africa, as in other low and middle-income countries, continues to fail in its basic duty to ensure that HWs do not violate women’s human rights. The health system at all levels is obliged to address mistreatment of women and hold every HW accountable for bringing change.

To ensure women’s safety, it is necessary to understand and address factors that contribute to disrespect and abuse in maternity facilities, including poor management and supervision [6, 19], normalisation of disrespect and abuse by communities and in facilities [6], poor work environment and lack of equipment and poor infrastructure [19]. Improvements are required to health facilities so that conditions are in place to deliver quality maternal care, ensuring adequate supplies and equipment to allow nurses to do their job effectively. Nurses working in public health facilities play a pivotal role in providing quality maternity services but limited resources, high workloads and shortage of staff often limit their ability to do so [41, 42]. Management personnel and systems must be strengthened to ensure that abusive care is prevented, and a high standard of maternity care is provided in line with existing maternal health policies and guidelines. Regular and supportive supervision of labour ward staff is required to ensure that nurses respect patient’s rights and provide holistic, patient-centered care for all women. Health workers should receive appropriate training about human rights, and the rights of women during labour and childbirth. Further, professional bodies have neglected their role in holding their members accountable for failing to respect human rights.

However, these recommendations essentially constitute a ‘business as usual’ approach and to date these measures have failed to achieve any real improvement. We therefore suggest that to effectively address the catastrophic failure of the South African health system to ensure the safety of women in its care, stronger action is required to hold HWs, health facilities and the overall health system accountable. We suggest that this can be done in several ways, including immediately implementing the existing policy allowing birth companions, strengthening complaints processes in facilities, routinely monitoring labour ward practices and holding HWs accountable for poor practices, and lastly, the option of criminalizing abusive behavior should be considered to strengthen HW accountability.

There is good evidence of the benefits of receiving continuous one-to-one support during labour, which include reducing the duration of labour, the likelihood of cesarean section or instrumental delivery, and the proportion of mothers reporting a negative birth experience [43]. In addition, several studies have shown that women who had birth companions were less likely to experience disrespect and abuse during childbirth [44–46]. Support can be provided by a person of the mothers choosing for example her partner or a family member, or by a Doula. Doulas are trained companions who provide physical, social and emotional support to mothers during pregnancy, labour and delivery, and who have received some training but are not employed by the health facility [47]. Support from a Doula has been shown to be the most successful model of one-to-one support for improving outcomes [48]. Our participants suggested that birth companions could reduce abusive care by empowering women to challenge these practices, as well as suggesting that having another person observing nurses could in itself reduce unacceptable behavior. Implementing a policy of birth companions or doulas would provide a mechanism for accountability as well as protecting women from abuse and disrespect, and would also motivate health facilities to improve and renovate labour ward facilities to an acceptable standard.

Measuring the quality of care received during labour and delivery from women’s perspectives could provide a mechanism to monitor health facilities’ performance in eliminating disrespectful and abusive care. Creating and implementing an exit survey questionnaire to be completed by women before discharge would serve as a measurement of the care women received, create awareness of disrespect and abuse, and encourage women to make complaints. The Person-Centered Maternity Care scale provides a strong evidence-based framework that could be adapted as a monitoring tool to measure quality of care [16].

Very few women lay complaints about the service they received in health facilities despite complaints procedures being in place, thereby further normalising poor service and disrespect [39]. None of the women in our study spoke of the possibility of complaining, rather choosing to stay quiet and avoid asking questions. Several reasons for this have been suggested, including lack of channels for laying complaints, power differences between nurses and patients, lack of choice of alternative health facilities, lack of knowledge, and perceived lack of response to complaints [49]. Health facilities should ensure that the complaints system is strengthened, and women are actively informed and encouraged to practice their rights.

Criminalising abusive behaviours by HWs is an action that should be considered. Demanding legal accountability from perpetrators and health facilities that allow these practices to persist, would bring into focus and into the public domain the seriousness of the problem. Pickles (2015) identifies abusive and disrespectful behavior as a violation of human rights and argues that intentional abusive maternity care should be labelled as specific form of gender-based violence and perpetrators should be criminalised. Some countries in Latin America, including Venezuala and Mexico, have developed such a legal response, introducing the new concept called ‘obstetric violence’ to address mistreatment during labour and delivery with aim of highlighting the significance of the problem [11, 38, 50]. It has been suggested that obstetric violence be adopted in SA so that for perpetrators to be held legally accountable for their actions [38], but it is important that individual health providers are not held accountable for failures of the state to provide adequate resources. The quality of care in health facilities will continue to deteriorate and uptake of maternal health services will decrease if substantial measures are not implemented to curb the pervasiveness of abusive and disrespectful behavior.

## Conclusion

Robust and wide-ranging interventions are required to address abusive practices during labour and delivery including improving health facilities’ infrastructure, management and supervision of labour ward staff and development of HW skills. However, given the persistence of a problem that was first identified over 20 years ago, stronger interventions are now required to ensure that these practices are finally eliminated. These should include systems to hold individual perpetrators and health facilities accountable as well as the health system overall. Furthermore, criminalising perpetrators of abusive and disrespectful care in health facilities could be considered.

## Supporting information

S1 File FGD guide Journey with my baby. (DOCX)
