# Supplementary material for: Health workers’ disrespectful and abusive behaviour towards women during labour and delivery: A qualitative study in Durban, South Africa
Source: PLoS One. 2021 Dec 14;16(12):e0261204. doi: 10.1371/journal.pone.0261204 (PMC8670673; doi:10.1371/journal.pone.0261204)
Supplement: S1 File — (DOCX) [file pone.0261204.s001.DOCX]

**Journey with my baby- timeline activity**

In this activity, women will be asked to draw a timeline of their life from pregnancy until now (when the baby is a few months old). In the timeline, women will highlight times of change from pregnancy through childbirth and the first months of the child’s life, particularly returning to work.

**Required**

- Coloured pens
- Prestik
- Small pieces of card (15 per mother)
- Flip chart paper (one for each mother) prepare the papers with a line along the middle.

**Instructions for mothers**

There is a line drawn across the paper, this represents the time since you found out that you were pregnant up to today. At the start of the line is the day you found out you were pregnant, at the end of the line is today.

Think of the most important things that have happened over the time when you were pregnant, having your baby, caring for your baby and returning to work

Either write or do a drawing of each event on a small piece of card

Stick the pieces of card along the line to show when these important occasions happened

On completion of the timeline you will be asked to explain to the group the things that have happened to you and why you chose these occasions to draw on your timeline

**Discussion question:**

On completion of the activity pin each of the flipcharts to the wall or ask people to gather round so everyone can see and ask each mother in turn to explain her own timeline, why each event was important, and how it made them feel.

- Why did you chose this event as being important?
- How did you feel when this happened? Explain whether you felt happy or sad at the time this event happened and why.
- Did other people have a similar experience- describe how others felt when this happened.
